# Calcium State-Dependent Regulation of Epithelial Cell Quiescence by Stanniocalcin 1a

**DOI:** 10.3389/fcell.2021.662915

**Published:** 2021-04-09

**Authors:** Shuang Li, Chengdong Liu, Allison Goldstein, Yi Xin, Caihuan Ke, Cunming Duan

**Affiliations:** ^1^State Key Laboratory of Marine Environmental Science, Xiamen University, Xiamen, China; ^2^College of Ocean and Earth Sciences, Xiamen University, Xiamen, China; ^3^Department of Molecular, Cellular, and Developmental Biology, University of Michigan, Ann Arbor, MI, United States

**Keywords:** PAPP-A, IGFBP-5, IGF1 receptor, Akt, Tor, ionocytes, zebrafish

## Abstract

The molecular mechanisms regulating cell quiescence-proliferation balance are not well defined. Using a zebrafish model, we report that Stc1a, a secreted glycoprotein, plays a key role in regulating the quiescence-proliferation balance of Ca^2+^ transporting epithelial cells (ionocytes). Zebrafish *stc1a*, but not the other *stc* genes, is expressed in a Ca^2+^ state-dependent manner. Genetic deletion of *stc1a*, but not *stc2b*, increased ionocyte proliferation, leading to elevated body Ca^2+^ levels, cardiac edema, body swelling, and premature death. The increased ionocyte proliferation was accompanied by an increase in the IGF1 receptor-mediated PI3 kinase-Akt-Tor signaling activity in ionocytes. Inhibition of the IGF1 receptor, PI3 kinase, Akt, and Tor signaling reduced ionocyte proliferation and rescued the edema and premature death in *stc1a^–/–^* fish, suggesting that Stc1a promotes ionocyte quiescence by suppressing local IGF signaling activity. Mechanistically, Stc1 acts by inhibiting Papp-aa, a zinc metalloproteinase degrading Igfbp5a. Inhibition of Papp-aa proteinase activity restored ionocyte quiescence-proliferation balance. Genetic deletion of *papp-aa* or its substrate *igfbp5a* in the *stc1a^–/–^* background reduced ionocyte proliferation and rescued the edema and premature death. These findings uncover a novel and Ca^2+^ state-dependent pathway regulating cell quiescence. Our findings also provide new insights into the importance of ionocyte quiescent-proliferation balance in organismal Ca^2+^ homeostasis and survival.

## Introduction

Maintaining a pool of quiescent cells that can be rapidly reactivated upon appropriate stimulation is critical for tissue repair, wound healing, and regeneration. This is particularly critical for highly renewable tissues such as epithelia (Valcourt et al., 2012; Cheung and Rando, 2013; Coller, 2019). Dysregulation of the cell quiescence-proliferation balance can lead to human diseases such as cancer, autoimmune diseases, and fibrosis (Kitaori et al., 2009; Fiore et al., 2018). Recent studies in genetically tractable organisms suggest that the nutrient sensitive insulin/insulin-like growth factor (IGF)-PI3 kinase-AKT-mTOR signaling pathway plays a key role in regulating the cell quiescence-proliferation decision. Studies in *Drosophila* have shown that adult neural stem cells can be reactivated in response to dietary amino acids attributed to increased insulin release from neighboring glia cells (Britton and Edgar, 1998; Chell and Brand, 2010; Sousa-Nunes et al., 2011; Huang and Wang, 2018). Likewise, mouse genetic studies revealed that IGF2 plays a key role in reactivating hematopoietic stem cells (HSCs), neural stem cell, and intestinal stem cells (Venkatraman et al., 2013; Ferron et al., 2015; Ziegler et al., 2015; Ziegler et al., 2019). Activating mTOR signaling by deleting the Tsc gene in mouse HSCs stimulates their cell cycle re-entry and proliferation (Chen et al., 2008). Conversely, inhibition of mTOR signaling in mice preserved the long-term self-renewal and the hematopoietic capacity of HSCs (Chen et al., 2009). This regulation is not limited to adult stem cells. mTORC1 signaling has been shown to promote naïve T cells to exit quiescence and proliferate (Yang et al., 2013).

While the importance of the insulin/IGF-PI3 kinase-AKT-mTOR signaling pathway in quiescence-proliferation regulation has become evident, an outstanding question is how this central hormonal pathway is activated in such a cell type-specific manner. Recently, we have developed a zebrafish model, in which a population of quiescent epithelial cells, known as ionocytes or NaR cells, can be induced to reenter the active cell cycle (Dai et al., 2014; Liu et al., 2017). NaR cells take up Ca^2+^ from the surrounding aquatic habitat to maintain body Ca^2+^ homeostasis (Hwang, 2009; Yan and Hwang, 2019). While largely quiescent when zebrafish larvae are kept in Ca^2+^-rich embryo rearing media, these cells rapidly re-enter the active cell cycle and proliferate when Ca^2+^ is depleted or reduced from the media (i.e., low [Ca^2+^] stress) (Dai et al., 2014; Liu et al., 2017). As the case in *Drosophila* and mouse adult stem cells, NaR cell quiescence to proliferation transition is regulated by the cell type-specific activation of the IGF-PI3 kinase-Akt-Tor signaling (Dai et al., 2014; Liu et al., 2017). Further studies suggested that IGF binding protein 5a (Igfbp5a), a secreted protein capable of binding IGFs with high-affinity and regulating IGF bioavailability to its receptors, and its major proteinase, pregnancy-associated plasma protein-a (Papp-aa), are highly expressed in NaR cells (Liu et al., 2018; Liu et al., 2020). Genetic deletion of *igfbp5a*, *papp-aa*, or inhibition of Papp-aa-mediated Igfbp5a proteolytic cleavage all abolishes NaR cell reactivation and proliferation (Liu et al., 2018, 2020). These findings suggest that Papp-aa-mediated Igfbp5a proteolysis plays a key role in activating IGF signaling locally and in promoting NaR cell quiescence exit and proliferation. Further analyses showed that the Papp-aa-mediated Igfbp5a proteolysis is inhibited by a post-transcriptional mechanism under normal [Ca^2+^] conditions. This in turn promotes NaR cell quiescence (Liu et al., 2020). The molecular nature of this [Ca^2+^]-dependent mechanism, however, is unknown.

Stanniocalcin 1 (Stc1) is a dimeric glycoprotein originally discovered from the corpuscles of Stannius (CS) in teleost fish in the 1960s (Wagner and Dimattia, 2006). Early studies showed that surgical removal of CS led to elevated calcium uptake, increased blood calcium level, and the appearance of kidney stones in fish (Fontaine, 1964; Pang, 1971; Fenwick, 1974), leading to the notion that CS contains a hypocalcemic hormone. The active CS component was purified and named as Stc1 (Wagner and Dimattia, 2006; Yeung et al., 2012). Mature Stc1 contains 11 conserved cysteine residues and a N-linked glycosylation site. The first 10 cysteines form intramolecular disulfide bridges and the 11th cysteine forms a disulfide bond linking the two monomers, which stabilizes the functional dimer (Yamashita et al., 1995). It is now clear that zebrafish genome contains 4 *stc* genes (Schein et al., 2012) and they are called by different names in the literature and in different databases. Hereafter, they will be referred as *stc1a, stc1b*, *stc2a*, and *stc2b* following Schein et al. (2012). In good agreement with the notion that Stc1 is a hypocalcemic hormone, forced expression by mRNA injection and morpholino-based knockdown of Stc1 in zebrafish altered Ca^2+^ uptake and changed NaR cell number, although these manipulations also changed the uptake of other ions as well as other ionocyte types (Tseng et al., 2009; Chou et al., 2015).

Although Stc1 was originally discovered in fish and was considered a teleost fish-specific hormone for several decades, it is now clear that multiple *Stc/STC* genes are present in mammals including humans (Gagliardi et al., 2005; Wagner and Dimattia, 2006; Yeung et al., 2012). Humans have two *STC* genes, *STC1* and *STC2*. There is limited and conflicting evidence on whether STCs play any major role in calcium uptake in mammals (Gagliardi et al., 2005; Wagner and Dimattia, 2006; Yeung et al., 2012). Overexpressing human STC1 or STC2 in transgenic mice resulted in reduced body size (Johnston et al., 2010). Biochemical studies showed that STC1 and STC2 can bind to PAPP-A/A2 *in vitro* and inhibit PAPP-A/A2-mediated IGFBP4 and IGFBP5 proteolytic cleavage (Argente et al., 2017). Clinical studies suggested that human individuals carrying loss-of-function mutations in the *STC2* gene had greater adult height and this was linked to reduced PAPP-A activity and increased local IGF signaling activity (Marouli et al., 2017). These observations led us to hypothesized that one or more Stc proteins regulates NaR cell quiescence by inhibiting Papp-aa-mediated Igfbp5a proteolysis and suppress IGF signaling.

In this study, we report that the expression of *stc1a*, but not the other 3 *stc* genes, is regulated in a [Ca^2+^] state-dependent manner in zebrafish embryos. Genetic deletion of *stc1a* resulted in elevated IGF signaling in NaR cells and increased NaR cell proliferation, leading to increased body Ca^2+^ levels, body edema, and premature death. These phenotypes were rescued by double deletion of *stc1a* with *papp-aa* or *igfbp5a*. Moreover, inhibition of IGF signaling reduced NaR cell proliferation and rescued the edema and premature death in *stc1a^–/–^* fish.

## Materials and Methods

### Chemicals and Reagents

Chemical and molecular biology reagents were purchased from Fisher Scientific (Pittsburgh, PA) unless otherwise noted. BMS-754807 was purchased from Active Biochemicals Co. Batimastat and ZnCl_2_ were purchased from Sigma (St. Louis, MO, United States), MK2206 from ChemieTek (Indianapolis, IN), wortamannin and Rapamycin from Calbiochem (Gibbstown, NJ). The Phospho-Akt antibody was purchased from Cell Signaling Technology (Danvers, MA, United States) and restriction enzymes were purchased from New England BioLabs (Ipswich, MA, United States). Primers, TRIzol, M-MLV reverse transcriptase were purchased from Life Technologies (Carlsbad, CA, United States). Anti-Digoxigenin-AP antibodies was purchased from Roche (Basel, Switzerland). The pT3.Cas 9-UTRglobin vector was a gift from Dr. Yonghua Sun, Institute of Hydrobiology, Chinese Academy of Sciences.

### Experimental Animals

Zebrafish were maintained, crossed, and staged in accordance to standard guidelines (Kimmel et al., 1995; Westerfield, 2000). Embryos and larvae were raised at 28.5°C in standard E3 embryo medium. Three additional embryo media containing 0.2, 0.02, and 0.001 mM [Ca^2+^] were prepared as previously reported (Dai et al., 2014). To inhibit pigmentation, 0.003% (w/v) N-phenylthiourea was added to these solutions. The *Tg(igfbp5a:GFP)* and *igfbp5a^–/–^* fish lines were generated in previous studies (Liu et al., 2018, 2020). The *papp-aa^*p170/*^*^+^ fish were a kind gift from the Marc Wolman lab. The *stc2b*^+ /−^ fish (*stc2b*^*sa24026*^, ZIRC# ZL10776) were obtained from ZIRC. All experiments were conducted in accordance with the guidelines approved by the Institutional Committee on the Use and Care of Animals, University of Michigan.

### RT-qPCR

Total RNA was extracted from pooled zebrafish embryos and larvae. RNA was reverse-transcribed to cDNA using oligo(dT)18 primer and M-MLV (Promega). qPCR was performed using SYBR Green (Bio-Rad) on a StepONE PLUS real-time thermocycler (Applied Biosystems). PCR primers were designed based on the 4 zebrafish *stc* gene sequences obtained from the Ensemble database (Ensemble gene numbers are: *stc1a*, ENSDARG00000058476, *stc1b*, ENSDARG00000003303, *stc2a*, ENSDARG00000056680, and *stc2b*, ENSDARG00000102206). The expression level of a target gene transcript was normalized by β*-actin* mRNA or *18S* RNA levels. The primers used are: *stc1a*-qPCR-F: 5′-CCAGCTGCTTCAAAACAAACC-3′, *stc1a*- qPCR-R: 5′-ATGGAGCGTTTTCTGGCGA-3′, *stc1b*-qPCR-F: 5′-CCAAGCCACTTTCCCAACAG-3′, *stc1b*-qPCR-R: 5′-ACCC ACCACGAGTCTCCATTC-3′, *stc2a*-qPCR-F: 5′-TATGGTCTT CCAGCTTCAGCG-3′, *stc2a*-qPCR-R: 5′-CGAGTAATGGCT TCCTTCACCT-3′, *stc2b*-qPCR-F: 5′-CACAAGAAAAGACTG TCTCTGCAGA-3′, *stc2b*-qPCR-R: 5′-GGTAGTGACATCTGG GACGG-3′, *papp-aa*-qPCR-F: 5′-AAAGAGGAGGGCGTTCA AG-3′, *papp-aa*-qPCR-R: 5′-TGCAGCGGATCACATTAGAG-3′ (Liu et al., 2020), *18s*-qPCR-F: 5′-AATCGCATTTGCCATCAC CG-3′, *18s*-qPCR-R: 5′-TCACCACCCTCTCAACCTCA-3′, β-*actin*-qPCR-F: 5′-GATCTGGCATCACACCTTCTAC-3′, β- *actin*-qPCR-R: 5′-CCTGGATGGCCACATACAT-3′.

### Generation of *stc1a^–/–^* Fish Lines and Transient Knockdown of *stc1a* by CRISPR/Cas9

Two sgRNAs targeting the *stc1a* gene were designed using CHOPCHOP^1^. Their sequences are: *stc1a*-sgRNA-1, 5′-GCAGA GCGCCATTCAGACAG-3′ and *stc1a*-sgRNA-2, 5′-GCAGAT CTCGTGCATGCCGT-3′. Mixed sgRNA (30–40 ng/μl) and Cas 9 mRNA (200–400 ng/μl) were injected into *Tg(igfbp5a:GFP)* embryos at the 1-cell stage (Xin and Duan, 2018). A subset of injected F0 embryos was used to identify indels. DNA was isolated from individual embryos and analyzed by PCR followed by hetero-duplex assays (HRMA) as reported (Liu et al., 2018). For transient knockout experiments, the remaining injected F0 embryos were raised in E3 embryo medium to 3 dpf and transferred to the intended embryo medium from 3 to 5 dpf as previously reported (Dai et al., 2014). To generate stable *stc1a^–/–^* fish lines, injected F0 embryos were raised to adulthood and crossed with *Tg(igfbp5a:GFP)* or wild-type fish. F1 fish were raised to adulthood and genotyped. After confirming indels by DNA sequencing, the heterozygous F1 fish were intercrossed to generate F2 fish.

### Genotyping

Genomic DNA was isolated from individual or pooled fish as reported (Liu et al., 2018). HRMA was performed to genotype *igfbp5a^–/–^*, *papp-aa^–/–^* fish and siblings following published methods (Liu et al., 2018, 2020). The *stc1a* mutant fish genotyping was performed by PCR using the following primers: *stc1a*-gt-F, 5′-TGAAAACCACTGCCTTAAATTG-3′, *stc1a*-gt-R, 5′-GTAGCTCTACCGATCCCAAATG-3′. The progenies of *stc2b*^+ /−^ intercrosses were genotyped by direct DNA sequencing.

### Morphology Analysis

Body length, somite number and head trunk angles were measured as described (Kamei et al., 2011; Liu et al., 2018). The bright-field images were acquired using a stereomicroscope (Leica MZ16F, Leica, Wetzlar, Germany) equipped with a QImaging QICAM camera (QImaging, Surrey, BC, Canada).

### Total Body Ca^2+^ Assay

The sample preparation was carried out as reported (Elizondo et al., 2010). Briefly, fish larvae were anesthetized using MS-222. In each group, 35 zebrafish larvae were pooled and washed twice with deionized water, and dried at 65°C for 60 min. Next, 125 μl 1M HCl was added to each tube and incubated overnight at 95°C with shaking. Samples were centrifuged and the supernatant was collected. Total calcium content was measured using a commercial kit (ab102505, Abcam, United States) following the manufacturer’s instruction.

### Live Imaging and Microscopy

NaR cells were quantified as previously reported (Liu et al., 2017). Briefly, embryos and larvae were anesthetized with MS-222. Larvae were mounted and subjected to fluorescent imaging using the Leica MZ16F stereo microscope. Image J was used for image analysis and data quantification.

### Immunostaining and Whole Mount *in situ* Hybridization

Whole mount immunostaining and *in situ* hybridization were performed as described previously (Dai et al., 2014). Briefly, zebrafish larvae were fixed in 4% paraformaldehyde and permeabilized in methanol. They were incubated overnight with the phospho-Akt antibody in 4°C, followed by incubation with an anti-rabbit HRP antibody (Jackson ImmunoResearch, West Grove, PA, United States) and nickel-diaminobenzidine staining. For *in situ* hybridization, *igfbp5a* mRNA signal was detected using a digoxigenin (DIG)-labeled antisense riboprobe. Larvae were incubated in an anti-DIG-AP antibody or dinitrophenol following published methods (Dai et al., 2014).

### Drug Treatment

All drugs used in this study, except ZnCl_2_, were dissolved in DMSO and further diluted in double-distilled water. ZnCl_2_ was dissolved in distilled water. Zebrafish larvae were treated with ZnCl_2_, batimastat, BMS-754807, and other drugs from 3 to 5 dpf (Dai et al., 2014; Liu et al., 2017). Drug solutions were changed daily. 5 dpf larvae were then collected for immunostaining, *in situ* hybridization or fluorescent imaging.

### Statistical Analysis

Statistical tests were carried out using GraphPad Prism 8 software (GraphPad Software, Inc., San Diego, CA). Values are shown as means ± SEM. Statistical significance between experimental groups was performed using unpaired two-tailed *t*-test, Chi-square test, long-rank test and one-way ANOVA followed by Tukey’s multiple comparison test. Statistical significances were accepted at *P* < 0.05 or greater.

## Results

### The Expression of *stc1a* Gene Is Regulated by Ca^2+^ Levels

**FIGURE 1:**
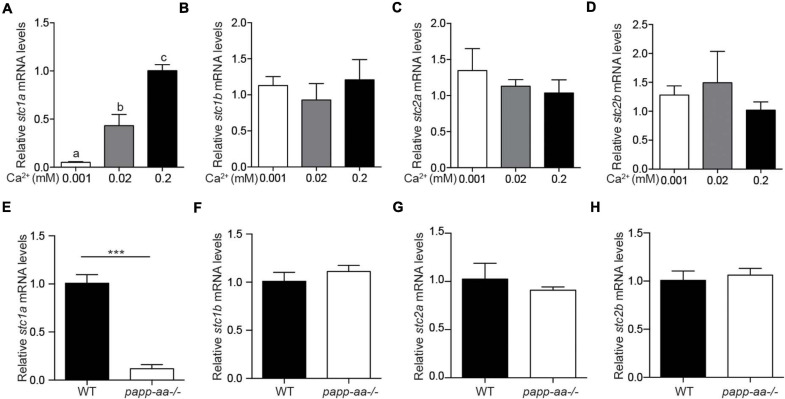
The expression of *stc1a*, but not other *stc* genes, is regulated by Ca^2+^ levels. **(A–D)** Wild-type zebrafish embryos were raised in embryo media containing the indicated Ca^2+^ concentration until 3 days post fertilization (dpf). The mRNA expression levels of *stc1a* **(A)**, *stc1b* **(B)**, *stc2a* **(C)**, and *stc2b* **(D)** were determined by qRT-PCR and normalized by β-actin mRNA levels. Data shown are from 3 independent experiments, each containing 10–15 embryos/group. In this and all subsequent figures, data shown are Mean ± SEM unless stated otherwise. Different letters indicate significant differences between groups by one-way ANOVA followed by Tukey’s multiple comparison test (*P* < 0.05) unless stated otherwise. **(E–H)** Zebrafish of the indicated genotypes were raised in E3 embryo medium until 5 dpf. The mRNA levels of *stc1a* **(E)**, *stc1b* **(F)**, *stc2a* **(G)**, and *stc2b* **(H)** were measured, normalized, and shown. Data shown are from 3 independent experiments, each containing 10–15 larvae/group. ***P < 0.001 by unpaired two-tailed *t*-test.

To determine whether changing Ca^2+^ levels affect *stc* expression, zebrafish embryos were raised in embryo media containing varying concentrations of [Ca^2+^]. As shown in Figures 1A–D, the levels of *stc1a* mRNA, but not those of *stc1b, stc2a, and stc2b*, were increased with increasing [Ca^2+^] levels. Similar results were obtained in larval fish (Supplementary Figure 1A). Next, we used *papp-aa^–/–^* and *trpv6^–/–^* mutants to investigate the effect of body Ca^2+^ levels. Both mutant fish suffer from severe calcium deficiency and had greatly reduced body Ca^2+^ levels compared to their siblings (Xin et al., 2019; Liu et al., 2020). The levels of *stc1a* mRNA were significantly lower in the *papp-aa^–/–^* mutant fish compared to those of the wildtype siblings (Figure 1E). The mRNA levels of *stc1b, stc2a*, and *stc2b* were comparable between *papp-aa^–/–^* and wild-type fish (Figures 1F–H). Similarly, the *stc1a* mRNA level in the calcium deficient *trpv6^–/–^* mutant fish was significantly lower compared to those of the siblings (Supplementary Figure 1B). These data suggest that the expression of *stc1a*, but not the other *stc* genes, is regulated in a [Ca^2+^]-dependent manner in zebrafish embryos.

### Genetic Deletion of *stc1a* Leads to Elevated NaR Cell Proliferation, Increased Body Ca^2+^ Content, Body Swelling, and Premature Death

**FIGURE 2:**
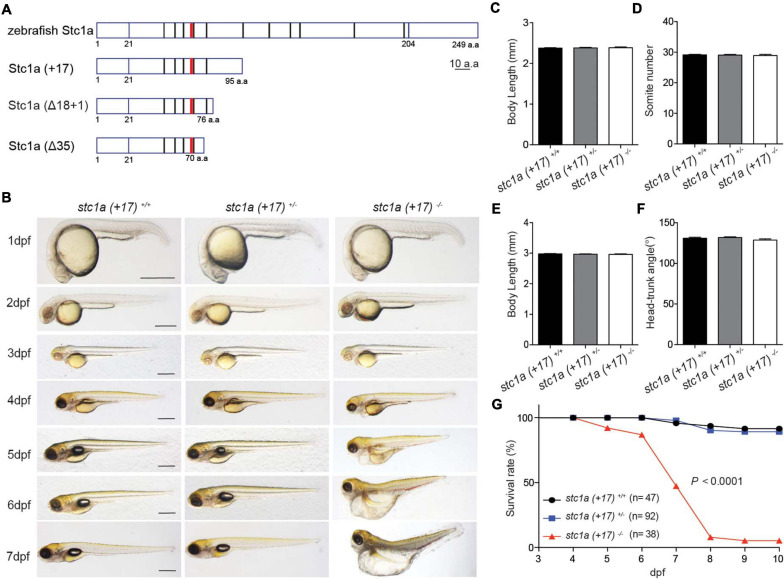
Genetic deletion of *stc1a* results in cardiac edema, body swelling, and premature death. **(A)** Schematic diagram of Stc1a protein and the indicated mutants. The N-linked glycosylation site and conserved cysteine residues are shown by red and black bars, respectively. **(B)** Morphology of fish of the indicated genotypes at the indicated stages. Lateral views with anterior to the left and dorsal up. Scale bar = 0.5 mm. **(C–F)** Body length, somite number, and head-trunk angle of the indicated genotypes at 24 hpf **(C,D)** and 48 hpf **(E,F)**. *n* = 15–42 fish/group. **(G)** Survival curves. Progenies of *stc1a* (+*17)^+/–^* intercrosses were raised in E3 embryo medium. Dead embryos were collected daily and genotyped individually. The survival curves of indicated genotypes and the total fish numbers are shown. *P* < 0.0001 by log-rank test.

To determine the *in vivo* function of Stc1a, three *stc1a* mutant fish lines, i.e., *stc1a* (+*17), stc1a* (Δ*18* + *1)*, and *stc1a* (Δ*35)*, were generated using CRISPR/Cas 9 (Supplementary Figure 2A). All 3 mutants are predicted to be null mutations (Figure 2A). Progenies of *stc1a* (+*17)^+ /−^* intercrosses, which were a mixture of homozygous, heterozygous, and wild type embryos, were raised and analyzed in a double-blind fashion. No difference in the body size, development speed, and gross morphology was detected among the 3 genotypes until 3 dpf (Figures 2B–F). At 4 dpf, many *stc1a* (+*17)^–/–^* mutant fish displayed cardiac edema (Figure 2B). The cardiac edema phenotype became more pronounced at 5 dpf. In addition, mutant fish had no inflated swim bladder (Figure 2B). By 6 and 7 dpf, mutant fish showed severe body swelling and death rate increased (Figures 2B,G). Similar results were obtained in *stc1a* (Δ*18* + *1)^–/–^* fish (Supplementary Figures 2B–G). In comparison, *stc2b^–/–^* fish showed no morphological defects or premature death (Supplementary Figure 3). These findings suggest that while Stc1a does not affect embryonic patterning and growth, it is essential for larval survival.

**FIGURE 3:**
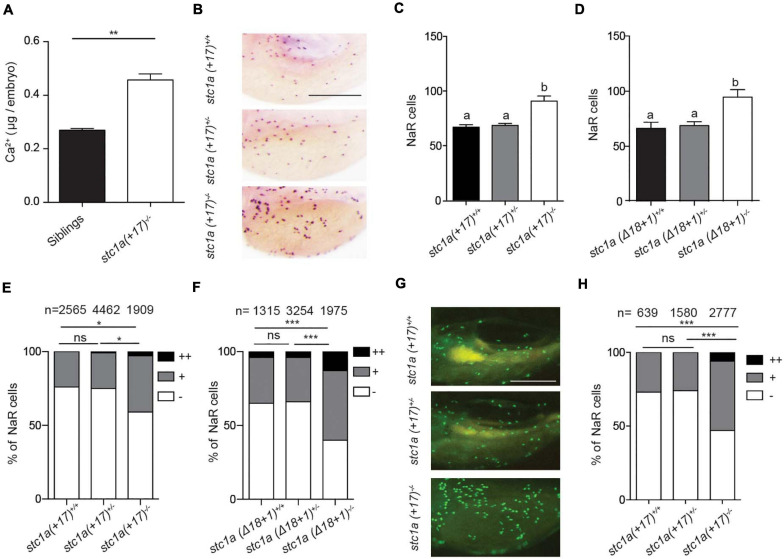
Genetic deletion of *stc1a* results in elevated body Ca^2+^ content and increased NaR cell proliferation. **(A)** Total body Ca^2+^ content in 5 dpf zebrafish larvae of the indicated genotypes. Data shown are from 3 independent experiments, each containing 35 larvae/group. **P < 0.01 by unpaired two-tailed *t*-test. **(B–D)** Progenies of *stc1a* (+*17)^+/–^* intercrosses **(C)** or progenies of *stc1a* (Δ*18* + *1)^+/–^* intercrosses **(D)** were raised in the E3 embryo medium to 5 dpf. NaR cells were detected by *in situ* hybridization using an *igfbp5a* cRNA probe. After NaR cells were visualized and quantified in each fish, fish were genotyped individually. Representative images are shown in **(B)** and quantified data in **(C,D)**. Scale bar = 0.2 mm. *n* = 33–70 larvae/group **(C)** and 19–46 larvae/group **(D)**. **(E,F)** NaR cells in 5 dpf larvae of the indicated genotypes were scored following a published proliferation scoring index (Liu et al., 2018). Cells that divided 0, 1, or 2 times were scored as -, +, and ++. *P < 0.05 and ***P < 0.001 by Chi-square test. Total number of cells is shown above the bar. **(G,H)** Progenies of *stc1a* (+*17)^+/–^; Tg (igfbp5a: GFP)* intercrosses were raised in E3 embryo medium until 5 dpf. GFP-expressing NaR cells were scored as described in **(E)**. Representative images are shown in **(G)** and quantified results in **(H)**. Scale bar = 0.2 mm. ***P < 0.001 by Chi-square test.

Because the known role of fish Stc1 in epithelial Ca^2+^ uptake and body Ca^2+^ balance, we measured total body Ca^2+^ levels. Measuring blood Ca^2+^ levels in zebrafish embryos/larvae is not feasible because of their tiny size. Compared to the siblings, the total body Ca^2+^ levels in *stc1a^–/–^* mutants were significantly greater (Figure 3A). Next, the possible changes in NaR cells were examined. For this, progenies of *stc1a* (+*17)^+/–^* intercrosses were subjected to *in situ* hybridization analysis using a specific NaR cell maker (Liu et al., 2017). *stc1a* (+*17)^–/–^* fish had significantly more NaR cells than their wild-type and heterozygous siblings (Figures 3B,C). Similar increases in NaR cell number were also observed in *stc1a* (Δ*18* + *1)^–/–^* (Figure 3D) and *stc1a* (Δ*35)^–/–^* fish (Supplementary Figure 4A). In comparison, *stc2b^–/–^* deletion did not increase NaR cell number (Supplementary Figure 4B). Notably, NaR cells in the *stc1a^–/–^* mutant fish were often observed as cell clusters, an indicator of newly divided cells (Figure 3B). Quantification results showed that the NaR proliferation index was greater in *stc1a* (+*17)^–/–^*, (Δ*18* + *1)^–/–^*, and *stc1a* (Δ*35)^–/–^* fish (Figures 3E,F and Supplementary Figure 4C). No change in NaR cell proliferation was detected in *stc2b^–/–^* fish (Supplementary Figure 4D). To investigate possible changes in NaR proliferation further, we generated a *stc1a* (+*17)^–/–^;Tg(igfbp5a:GFP*) fish line. The *Tg(igfbp5a:GFP)* fish is a reporter fish line, in which NaR cell proliferation can be visualized in real time in live fish larvae (Liu et al., 2017). A significantly higher NaR cell proliferation rate was detected in *stc1a* (+*17)^–/–^* fish compared with the siblings (Figures 3G,H). These findings suggest that genetic deletion of *stc1a* results in elevated NaR cell proliferation and higher body Ca^2+^ content.

### Stc1a Action in NaR Cells Requires Papp-aa and Igfbp5a

**FIGURE 4:**
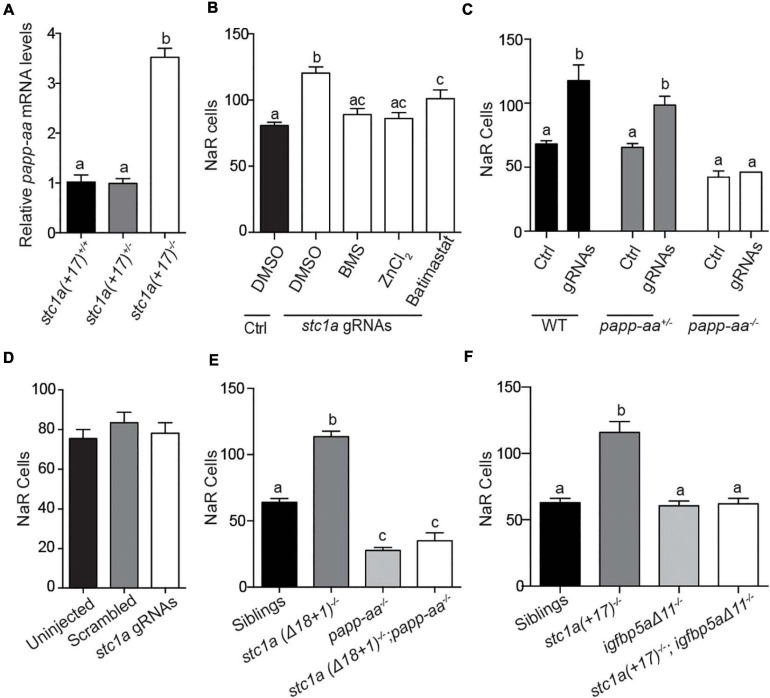
Papp-aa and Igfbp5a are indispensible for Stc1a action in NaR cells. **(A)** Embryos of the indicated genotypes were raised in E3 embryo medium until 5 dpf. The mRNA levels of *papp-aa* were measured and normalized. *n* = 15–17 larvae/group. **(B)** *Tg(igfbp5a: GFP)* embryos were injected with *stc1a* targeting gRNAs and Cas9 mRNA at the 1-cell stage. Embryos were raised in E3 embryo medium. The injected embryos were treated with BMS-754807 (BMS, 0.3 μM), ZnCl_2_ (8 μM), or Bastimastat (200 μM) from 3 to 5 dpf. NaR cells were quantified and shown. *n* = 20–39 larvae/group. **(C,D)** Progeny of *papp-aa^+/–^; Tg (igfbp5a: GFP)* intercrosses **(C)** or *igfbp5a^– /–^; Tg (igfbp5a: GFP)* intercrosses **(D)** were injected with *stc1a* targeting gRNAs and Cas9 mRNA. The injected embryos were raised and NaR cells were quantified at 5 dpf. Each larva was genotyped afterward. *n* = 5–19 larvae/group **(C)** and *n* = 39–43 larvae/group **(D)**. **(E,F)** Larvae of the indicated genotypes were raised in E3 embryo medium and NaR cells were quantified at 5 dpf. *n* = 9∼78 larvae/group **(E)** and *n* = 4∼34 larvae/group **(F)**.

Gene expression indicated higher *papp-aa* mRNA levels in *stc1a* (+*17)^–/–^* fish compared with their siblings (Figure 4A). Knockdown of Stc1a using gRNAs resulted in a significant increase in NaR cell number (Figure 4B). This increase was reduced by treatment with ZnCl_2_ or batimastat, two distinct Papp-aa inhibitors (Tallant et al., 2006; Liu et al., 2020; Figure 4B), suggesting Papp-aa-mediated proteolysis is critical. This action is IGF signaling-dependent since it was abolished by treatment with BMS-754807, an IGF1 receptor inhibitor (Figure 4B).

If Stc1a acts via suppressing the Papp-aa-mediated Igfbp5a proteolysis, then the loss of Stc1a should not affect NaR cell quiescence-proliferation balance in the absence of Papp-aa or Igfbp5a. Indeed, while knockdown of Stc1a resulted in significant increases in NaR cell proliferation in wild-type and heterozygous siblings, it did not have such an effect in *pappaa^–/–^; Tg (igfbp5a:GFP)* embryos (Figure 4C). Likewise, knockdown of Stc1a in *igfbp5a^–/–^; Tg (igfbp5a:GFP)* fish did not increase NaR cell proliferation (Figure 4D). This was tested further by generating stable double mutant fish. While permanent deletion of *stc1a* significantly increased NaR cell reactivation, it had no such effect in the *papp-aa^–/–^* background (Figure 4E) or in the *igfbp5a^–/–^* background (Figure 4F). These genetic data suggest that Stc1a promotes NaR cell quiescence in a Papp-aa and Igfbp5a-dependent manner.

### Stc1a Promotes NaR Cell Quiescence by Suppressing IGF Signaling in NaR Cells

**FIGURE 5:**
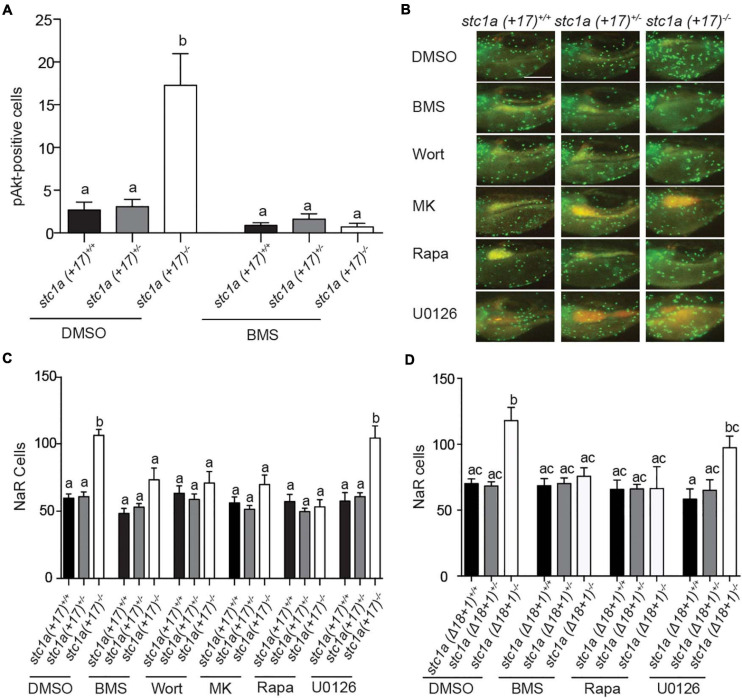
Stc1a promotes NaR cell quiescence by suppressing IGF-PI3 kinase-Akt-Tor signaling in NaR cells. **(A)** Progenies of *stc1a* (+ *17)^+/–^; Tg (igfbp5a: GFP)* intercrosses were raised in E3 embryo medium to 3 dpf and treated with DMSO or 0.3 μM BMS-754807 (BMS). Two days later, fish were fixed and phospho-Akt positive cells in the yolk sac region were detected by immunostaining. These fish were genotyped individually afterward. *n* = 14–47 larvae/group. **(B,C)** Progeny of *stc1a* (+ *17)^+/–^; Tg (igfbp5a: GFP)* intercrosses were raised in E3 embryo medium and treated with DMSO, 0.3 μM BMS-754807 (BMS), 0.06 μM Wortmannin (Wort), 8 μM MK2206 (MK), 5 μM Rapamycin (Rapa), or 10 μM U0126 from 3 to 5 dpf. After NaR cells were quantified, these fish were genotyped individually. Representative images **(B)** and quantified data are shown **(C)**. *n* = 7–35 larvae/group. Scale bar = 0.2 mm. **(D)** Progeny of *stc1a* (Δ*18* + *1)^+/–^; Tg (igfbp5a: GFP)* intercrosses were raised in E3 embryo medium to 3 dpf and treated with DMSO, 0.3 μM BMS-754807 (BMS), 5 μM Rapamycin (Rapa), or 10 μM U0126 from 3 to 5 dpf. NaR cells were quantified as described above. *n* = 4–28 larvae/group.

To determine whether local IGF signaling plays a role in elevated NaR cell proliferation in *stc1a^–/–^* fish, phospho-Akt immunostaining analysis was performed as previously reported (Dai et al., 2014). Compared with siblings, *stc1a* (+*17)^–/–^* larvae had significantly more phospho-Akt positive cells in the yolk sac region (Figure 5A). This increase was abolished by the addition of the IGF1 receptor inhibitor BMS-754807 (Figure 5A). Moreover, BMS-754807 treatment reduced NaR cell number in *stc1a* (+*17)^–/–^* larvae to that of the siblings (Figures 5B,C). Likewise, treatments with the PI3 kinase inhibitor wortamannin, Akt inhibitor MK2206, and Tor inhibitor rapamycin all decreased NaR cell proliferation (Figures 5B,C). The addition of the MEK inhibitor U0126 had no such effect level (Figures 5B,C). Similar results were obtained with *stc1a* (Δ*18* + *1)^–/–^* fish (Figure 5D). These data suggest that genetc deletion of *stc1a* increases NaR cell proliferation via activating the IGF1 receptor-mediated PI3 kinase-Akt-Tor signaling in NaR cells. Therefore, Stc1a promotes NaR cell quiescence state by suppressing local IGF signaling.

### The Body Edema and Premature Death in *stc1a^–/–^* Fish Are Rescued by Perturbing the Papp-aa-Igfbp5a-IGF Signaling Axis

**FIGURE 6:**
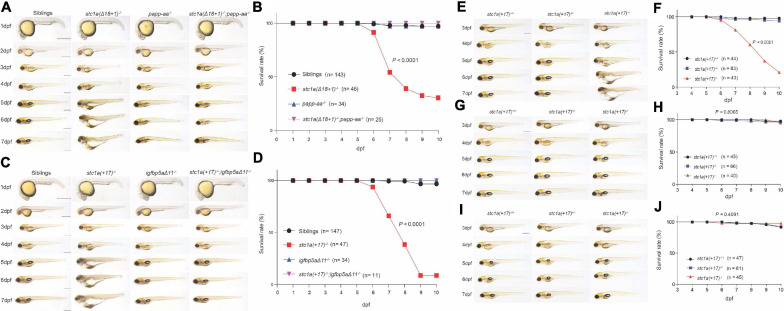
Perturbation of the Papp-aa-Igfbp-a-IGF signaling loop rescues the body edema and premature death in *stc1a^– /–^* fish. **(A,C)** Gross morphology of fish of the indicated genotypes at the indicated stages. Lateral views with anterior to the left and dorsal up. Scale bar = 0.5 mm. **(B,D)** Survival curves. Embryos were raised in E3 embryo medium. Dead embryos were collected daily and genotyped individually. The survival curves of indicated genotypes and the total fish numbers are shown. *P* < 0.0001 by log-rank test. **(E–J)** Progeny of *stc1a* (+ *17)^+/–^* intercrosses were raised in E3 embryo media containing DMSO **(E,F)**, 0.3 μM BMS-754807 **(G,H)** or 5 μM Rapamycin **(I,J)** starting from 3 dpf until the indicated time. Representative morphological views of the indicated genotypes at the indicated stages are shown in **(E,G,I)**. Scale bar = 0.5 mm. *P* < 0.0001 by log-rank test. Dead fish were collected daily and genotyped individually. The survival curves of the indicated genotypes and the total fish numbers are shown in **(F,H,J)**.

The cardiac edema, body swelling, and premature death phenotypes in *stc1a^–/–^* fish were unexpected. We postulated that these phenotypes may be related to the altered NaR cell quiescence-proliferation balance in the *stc1a^–/–^* mutant fish. We tested this idea using *stc1a^–/–^;papp-aa^–/–^* double mutant fish. While *stc1a^–/–^* fish developed cardiac and body edema, and had elevated death rate, *stc1a^–/–^;papp-aa^–/–^* double mutants did not display these phenotypes (Figure 6A). The survival curve of *stc1a^–/–^;papp-aa^–/–^* double mutant fish was similar to their wild-type and heterozygous siblings (Figure 6B). Likewise, permanent deletion of *igfbp5a* in the *stc1a^–/–^* background prevented the development of edema, swelling and premature death (Figures 6C,D). Finally, treatment of *stc1a^–/–^* fish with BMS-754807 eliminated the edema, body swelling and prevented the mutant fish from premature death (Figures 6E–H). Inhibition of Tor signaling had similar results (Figures 6I,J). The cardiac edema, body swelling, and premature death phenotypes in *stc1a^–/–^* fish occur due to elevated IGF signaling and NaR cell quiescence-proliferation imbalance.

## Discussion

Ca^2+^ is an essential ion and plays key roles in a wide range of biological processes. In zebrafish embryos, epithelial Ca^2+^ uptake is carried out by NaR cells (Liao et al., 2009; Yan and Hwang, 2019). NaR cells are functionally equivalent to human intestinal and renal epithelial cells and express major molecular components of the transcellular Ca^2+^ transport machinery, including the epithelial calcium channel Trpv6 (Hoenderop et al., 2005; Hwang, 2009). In the adult stage, NaR cells are distributed in the intestine, kidney, and gills (a major osmoregulation organ in fish). In the embryonic and larval stages, these cells are located on the yolk sac region (Hwang, 2009). Genetic deletion of *trpv6* resulted in severe Ca^2+^ deficiency and premature death in zebrafish (Xin et al., 2019). Epithelial Ca^2+^ uptake is regulated by a number of hormones, including parathyroid hormones, vitamin D, isotocin, cortisol, stanniocalcin 1, and IGF1 (Chou et al., 2011, 2015; Dai et al., 2014; Yan and Hwang, 2019). Calcium abundance/availability also changes epithelial Ca^2+^ uptake by affecting Trpv6 expression and NaR cell numbers (Liu et al., 2017; Yan and Hwang, 2019). We have previously reported that reducing or depleting Ca^2+^ from the embryo media (i.e., low Ca^2+^ stress) activates IGF signaling in NaR cells locally and stimulates pre-exiting NaR cells to re-enter the cell cycle and proliferate (Dai et al., 2014; Liu et al., 2017). In this cell-type specific regulation of IGF signaling, IGFBP5/Igfbp5a and its proteinase Papp-aa are key players (Liu et al., 2020; Liu et al., 2018). Under low [Ca^2+^] conditions, Papp-aa degrades Igfbp5a and releases IGFs to activate IGF signaling and promotes NaR proliferation. Under normal [Ca^2+^] conditions, however, the Papp-aa-mediated Igfbp5 proteolysis is inhibited and IGF signaling is suppressed in NaR cells. This promotes NaR cell quiescence (Liu et al., 2020). In this study, we provide genetic, cell biology, and pharmacological evidence suggesting that Stc1a functions as a calcium state-dependent regulator of IGF signaling in NaR cell quiescence and promotes NaR cell quiescence state. Mechanistically, Stc1a suppresses local IGF signaling by inhibiting Papp-aa-mediated Igfbp5a proteolytic cleavage.

Early reports showed that Stc1 expression is stimulated by increasing external [Ca^2+^] via the action of calcium-sensing receptor (CaSR), a G-protein coupled receptor which senses extracellular [Ca^2+^] levels. When kept in high [Ca^2+^] media, cultured rainbow trout CS cells increase Stc1 expression and secretion (Ellis and Wagner, 1995). This increase in Stc1 expression is mediated by CaSR (Radman et al., 2002). Similarly, zebrafish embryos raised in embryo media containing higher levels of [Ca^2+^] had greater levels of *stc1* mRNA compared to those raised in normal [Ca^2+^] media and this increase is mediated by CaSR (Kwong et al., 2014; Lin et al., 2014). We now understand that teleost fish and mammals have multiple STC/Stc genes. Whether all *stc* genes are regulated in a similar way and act redundantly is less clear. Additionally, whether Stc1 expression is regulated in a similar fashion by body calcium levels *in vivo* has not yet reported. The results of the present study addressed these questions. Our results suggested that the 4 *stc* genes are differentially regulated in zebrafish embryos. Increasing external [Ca^2+^] levels increased *stc1a* mRNA levels in a concentration-dependent manner. This effect is specific to *stc1a* and no such changes were found with *stc1b*, *stc2a*, and *stc2b*. The effects of the body Ca^2+^ levels were determined by comparing *stc* mRNA levels in calcium deficient *papp-aa^–/–^* fish and their siblings (Xin et al., 2019; Liu et al., 2020). The levels of *stc1a* mRNA were significantly reduced in the *papp-aa^–/–^* mutant fish compared to their siblings. Papp-aa is a zinc metalloproteinase involved in degrading Igfbps (Liu et al., 2020). Therefore, the reduced *stc1a* mRNA levels in *papp-aa^–/–^* fish is probably caused by the reduced body Ca^2+^ levels in *papp-aa^–/–^* fish rather than a direct effect of Papp-aa. This notion was further supported by the reduced *stc1a* mRNA level in the *trpv6^–/–^* mutant fish.

**FIGURE 7:**
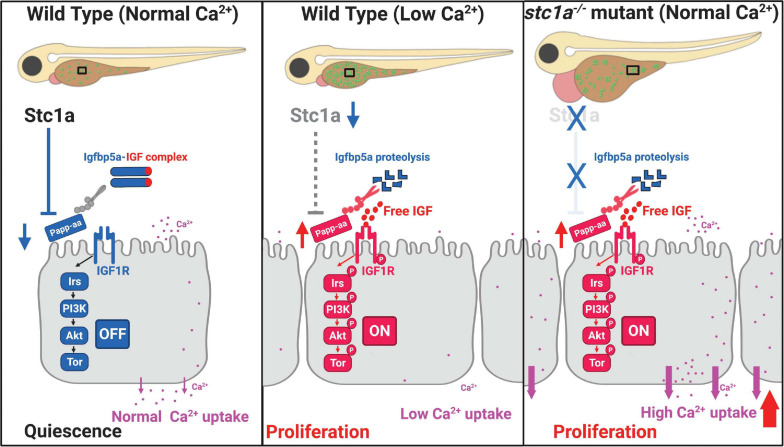
Proposed model of Stc1a function as a [Ca^2+^] state-regulated regulator of local IGF signaling and its role in epithelial cell quiescence-proliferation balance.

The first Stc1 protein was identified from bony fish in 1960s (Pang, 1973; Wagner and Dimattia, 2006; Yeung et al., 2012). While significant progress has been made in our understanding of Stc proteins over the past decades, the long-term *in vivo* function of fish Stc has not been elucidated due to the lack of a null fish model. Using CRISPR/Cas9, we generated several *stc1a^–/–^* zebrafish lines. Our genetic analysis results reveal that Stc1a is an essential protein. Loss of Stc1a leads to cardiac edema, body swelling, and premature death. Importantly, loss of *stc1a* resulted in elevated NaR cell proliferation and increased NaR cell number, suggesting Stc1a regulates NaR cell quiescence-proliferation balance. The action of Stc1a in NaR cells clearly involves local IGF signaling. Loss of Stc1a activated the IGF1 receptor-mediated Akt signaling in NaR cells. Inhibition of the IGF1 receptor, PI3 kinase, Akt, and Tor all reduced NaR cell proliferation in *stc1a^–/–^* mutant fish. The *papp-aa* mRNA levels were higher in the *stc1a^–/–^* mutant fish. In zebrafish embryos, *papp-aa* mRNA is highly expressed in NaR cells (Liu et al., 2020). The elevated *papp-aa* mRNA level in *stc1a^–/–^* mutant fish is likely an indirect result of increased NaR cell number. We investigated the functional relationship between Stc1a and Papp-aa using genetic and pharmacological approaches. While transient knockdown of Stc1a increased NaR cell prolifeartion in wild-type fish, it did not have such effect in *papp-aa^–/–^* mutant fish. Likewise, double deletion of *papp-aa* and *stc1a* reduced NaR cell proliferation. Finally, pharmacological inhibition of Papp-aa-mediated proteolysis reduced the NaR cell proliferation in *stc1a^–/–^* mutant fish to the sibling levels, suggesting that Papp-aa and its proteolysis are required for Stc1a action in NaR cells. In further support of this conclusion, knockdown or genetic deletion of *igfbp5a*, which encodes the major Papp-aa substrate Igfbp5a, abolished the NaR cell proliferation in *stc1a^–/–^* mutant fish. Based on these findings, we propose that Stc1a regulates NaR cell quiescence-proliferation balance in a [Ca^2+^] state-dependent manner (Figure 7). Under normal [Ca^2+^] conditions, Stc1a is expressed and Stc1a inhibits Papp-aa-mediated Igfbp5a proteolysis. The intact Igfbp5a binds to and sequesters IGFs in the Igfbp5a/IGF complex. This suppresses IGF signaling and promotes NaR cell quiescence (Figure 7, left panel). Under low [Ca^2+^] conditions, Stc1a expression is reduced and this inhibitory loop is inactivated and Papp-aa-mediated Igfbp5a proteolysis increased. The free IGFs binds to the IGF1 receptor and activates the IGF1 receptor-mediated PI3 kinase-Akt-Tor signaling in NaR cells. This stimulates NaR cell proliferation and increases NaR cell number. Despite the increased NaR cell number, the body calcium levels are low because the external Ca^2+^ is depleted (Figure 7, middle panel). In the *stc1a^–/–^* mutant fish, Stc1a is absent and Papp-aa is active. Papp-aa proteolytically cleaves Igfbp5a and releases IGFs from the complex to activate IGF-1 receptor-mediated signaling and promotes NaR cell proliferation. Increased NaR cell number results in elevated Ca^2+^ uptake and this in turn leads to cardiac edema and body swelling (Figure 7, right panel). While our conclusion is based on findings made in zebrafish, available evidence indicates that this regulatory loop is conserved in mammals. Human STC1 has been shown to bind to PAPP-A and inhibit PAPP-A proteolytic cleavage of IGFBP4 and IGFBP5 *in vitro* (Kløverpris et al., 2015). A recent study in mice suggested that mesenchymal stromal cells secrete STC1 to suppress HSC reactivation and proliferation (Waclawiczek et al., 2020).

It is worth to note that the *stc1a^–/–^* mutant fish phenotypes reported in the present study differ considerably from those reported in Stc1-null mice. Stc1-null mice grew and reproduced normally with no notable anatomical abnormalities (Chang et al., 2005). There are several possible explanations for the differences. Published studies suggest that mammalian STC-1 does not appear to play a major role in Ca^2+^ uptake nor does it act as an endocrine factor (Wagner and Dimattia, 2006; Yeung et al., 2012). Mammals do not have CS glands. The *STC1* gene is expressed in many tissues in mammals and acts mainly in a paracrine and autocrine fashion (Yeung et al., 2012). Another key difference is the habitats. Unlike the terrestrial mammals, zebrafish live in hypoosmotic aquatic habitats (Evans, 2008). Zebrafish must constantly uptake ions such as Ca^2+^ from the surrounding habitat and continously remove osmotic water by secreting a large volume of diluted urine to maintain the body osmolarity (Evans, 2008). We speculate that the increased NaR cell number and elevated epithelial Ca^2+^ uptake in *stc1a^–/–^* mutant fish led to increased osmotic water in the fish body, resulting in cardiac dema and body swelling. This speculation is supported by the fact that when the elevated NaR cell proliferation in *stc1a^–/–^* mutant fish was blocked by inhibiting IGF signaling, the body edema and premature death phenotypes were alleviated. Moreover, genetic deletion of *igfbp5a* or *papp-aa* in the *stc1a^–/–^* background reduced NaR cell proliferation and prevented the cardiac edema, body swelling, and premature death phenotypes. It is also possible that the higher levels of body Ca^2+^ may play a direct role in heart development and/or cardiac muscle function. However, we did not notice significant changes in hear development in *stc1a^–/–^* embryos. In fact, the *stc1a^–/–^* mutant fish are indistinguishable from the siblings until 3 dpf. The cardiac edema began to appear around 4 pdf and became more prevalent thereafter. Future studies are needed to clarify whether the higher levels of Ca^2+^ play ant direct role in cardiac muscle.

## Conclusion

In conclusion, our analyses reveal a previously unrecognized role of Stc1a as a [Ca^2+^] state-dependent epithelial cell quiescence regulator. Stc1a regulates epithelial cell quiescence-proliferation balance by suppressing the local Papp-aa-Igfbp5a-IGF signaling loop. Our study also sheds new light on the functional importance of ionocyte quiescence-proliferation balance in organismal Ca^2+^ homeostasis and survival. Zebrafish NaR cells are functionally and molecularly similar to human renal epithelial cells. In mammals, dysregulation of IGF signaling has been implicated in abnormal kidney growth. Following unilateral nephrectomy, for example, the contralateral kidney shows compensatory growth in an IGF dependent manner (Rosendahl et al., 1992). Renal expression of IGF-1, IGF1 receptor, IGFBP4, IGFBP5, and PAPP-A are all increased in an autosomal dominant polycystic disease (ADPKD) mouse model (Kashyap et al., 2020). A recent study reported that PAPP-A knockout significantly decreased cyst development and improved kidney injury response in the ADPKD mice (Kashyap et al., 2020). STC1 has been identified as one of the chronic kidney disease genes by genome-wide association studies (Böger and Heid, 2011). Future studies will be needed to elucidate whether STC1 plays a similar role in regulating mammalian renal cell quiescence-proliferation balance via locally expressed PAPP-A, IGFBPs, and IGF signaling.

## Data Availability Statement

The raw data supporting the conclusions of this article will be made available by the authors, without undue reservation.

## Ethics Statement

The animal study was reviewed and approved by the Institutional Committee on the Use and Care of Animals, University of Michigan.

## Author Contributions

CD conceived the study. CD and SL designed the study and wrote the manuscript. SL, CL, AG, and YX performed the study. SL analyzed the data. CD and CK provided supervision. All authors read the manuscript.

## Conflict of Interest

The authors declare that the research was conducted in the absence of any commercial or financial relationships that could be construed as a potential conflict of interest.

## Footnotes

1 http://chopchop.cbu.uib.no
