# Supplementary material for: Calcium State-Dependent Regulation of Epithelial Cell Quiescence by Stanniocalcin 1a
Source: Front Cell Dev Biol. 2021 Apr 9;9:662915. doi: 10.3389/fcell.2021.662915 (PMC8063699; doi:10.3389/fcell.2021.662915)
Supplement: Supplementary file 11 [file Data_Sheet_1.PDF]

**Supplemental Materials**

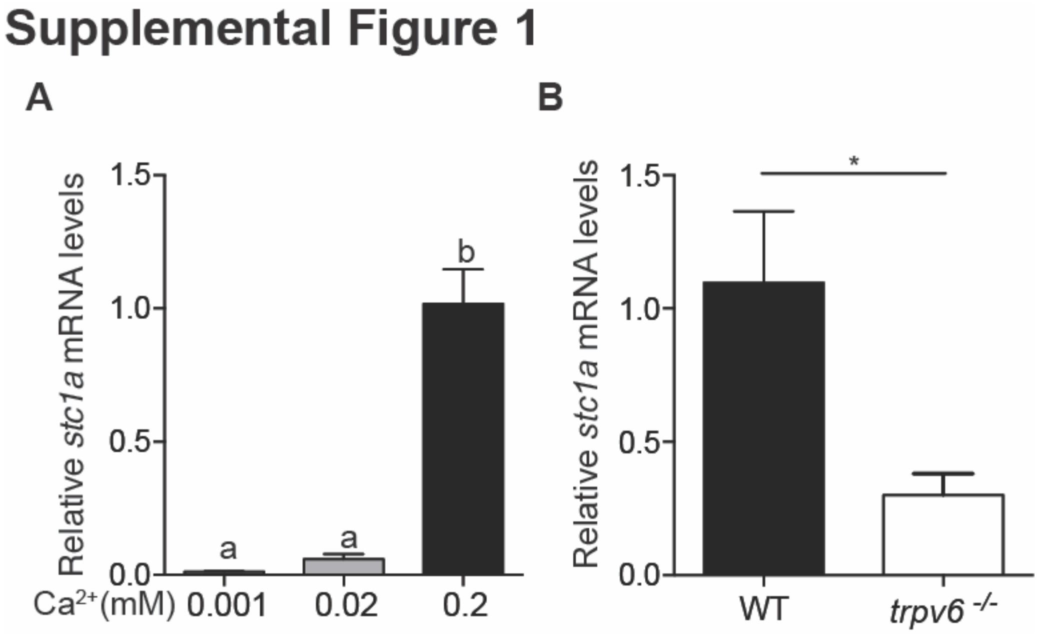


Supplemental Figure 1. Regulation of *stc1a* expression by $Ca^{2+}$ states in larval zebrafish. (A) Wild-type zebrafish were raised in embryo media containing the indicated $Ca^{2+}$ concentration from 5 to 7 dpf. The mRNA expression levels of *stc1a* were determined by qPCR and normalized by β-actin mRNA levels. Data shown are from 3 independent experiments, each containing 10-15 larvae/group. Data shown are are Mean ± SEM from 3 independent experiments, each containing 10-15 larvae/group. Different letters indicate significant differences between groups by one-way ANOVA followed by Tukey's multiple comparison test ($P < 0.05$). (B) Zebrafish embryos of the indicated genotypes were raised in E3 embryo medium until 5 dpf. The level of *stc1a mRNA* was measured and shown. Data shown are from 3 independent experiments, each containing 10-15 larvae/group. *, $P < 0.05$ by unpaired two-tailed t test.

## Supplemental Figure 2

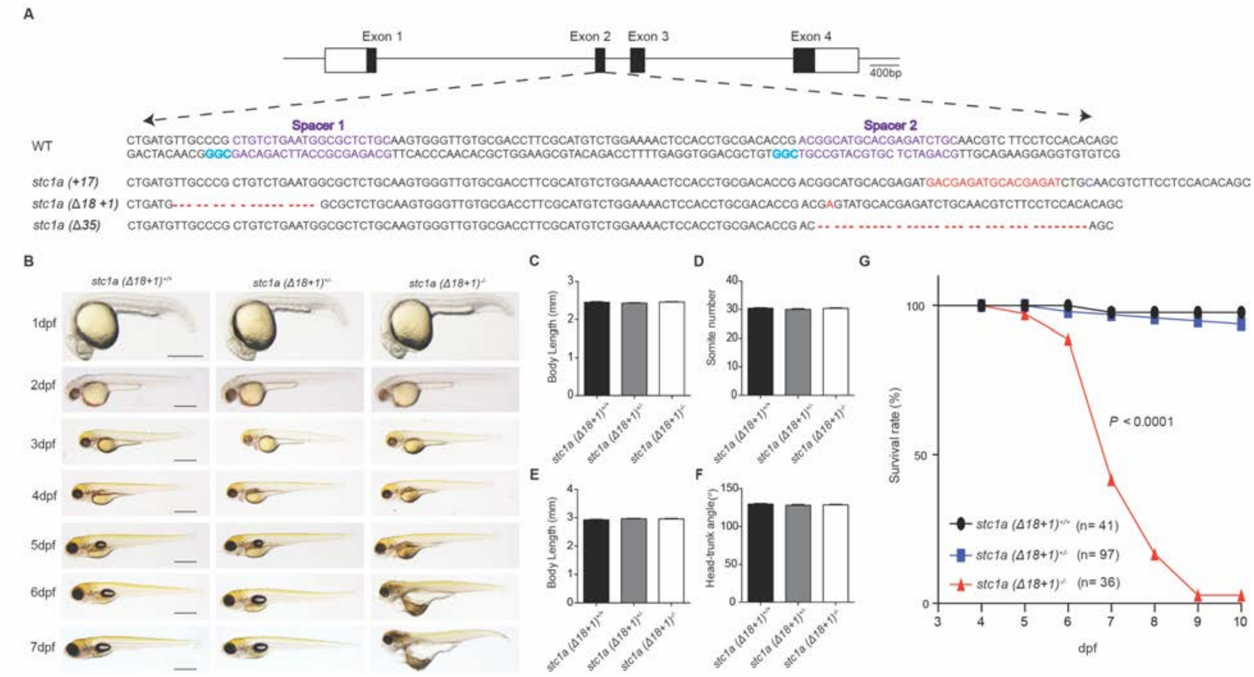


Supplemental Figure 2. Genetic deletion of Stc1a results in cardiac edema, body swelling, and premature death. (A) Schematic diagram of *stc1a* and the CRISPR/Cas9 targeting sites. Exons are represented as boxes and introns as lines. Open and filled boxes are untranslated and protein coding regions, respectively. The DNA sequence of wild-type fish (WT) and the indicated mutants are shown. The PAM motif is highlighted in blue. The deletion or insertion is highlighted in red. (B) Gross morphology of wild-type (WT), *stc1a (Δ18+1)*$^{+/-}$ and *stc1a (Δ18+1)*$^{-/-}$ fish at the indicated stages. Representative images are shown. Scale bar = 0.5 mm. (C-F) Body length, somite number, and head trunk angle of wild-type (WT), *stc1a (Δ18+1)*$^{+/-}$ and *stc1a (Δ18+1)*$^{-/-}$ fish were measured at 24 hpf (C, D) and 48 hpf (E, F). n = 14-39 larvae/group. (G) Survival curves. Progeny of *stc1a (Δ18+1)*$^{+/-}$ intercrosses were raised in E3 embryo medium. Dead embryos were collected daily and genotyped individually. The survival curves of indicated genotypes and the total fish numbers are shown. *P* < 0.0001 by log-rank test.

## Supplemental Figure 3

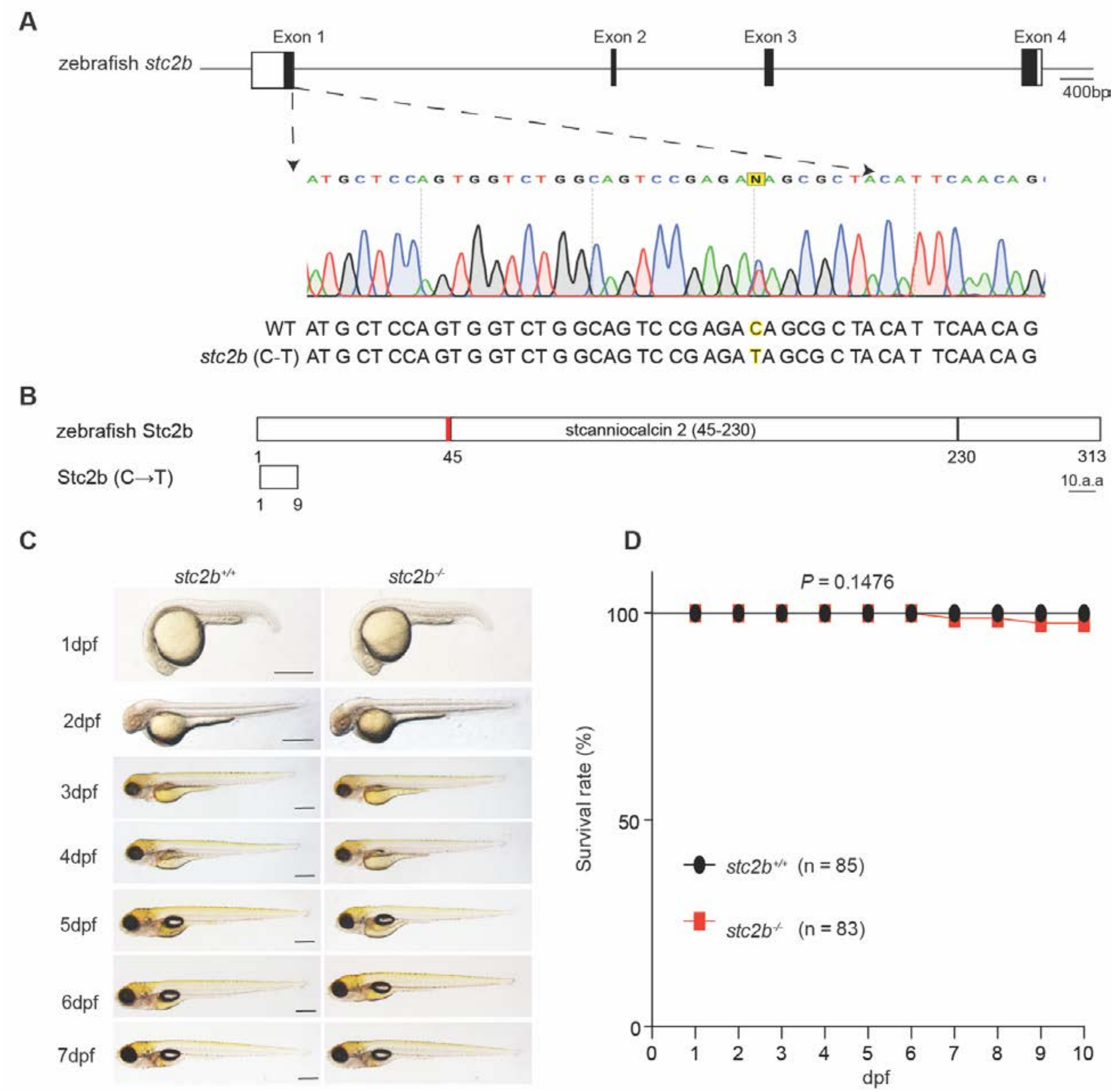


Supplemental Figure 3. (A) Schematic diagram of the *stc2b* and *stc2b*$^{-/-}$ mutant sequence. Exons are represented as boxes and introns as lines. Open and filled boxes are untranslated and protein coding regions, respectively. DNA sequence of *stc2b* and *stc2b*$^{-/-}$. The altered base is shown in yellow. (B) Schematic diagram of Stc2b protein and its mutants. The N-linked glycosylation site is shown by the red bar. (C) Gross morphology of wild-type and *stc2b*$^{-/-}$ fish at the indicated stages. Representative images are shown. Scale bar = 0.5 mm. (D) Survival curves of wild-type and *stc2b*$^{-/-}$ fish. The total fish numbers are shown.

## Supplemental Figure 4

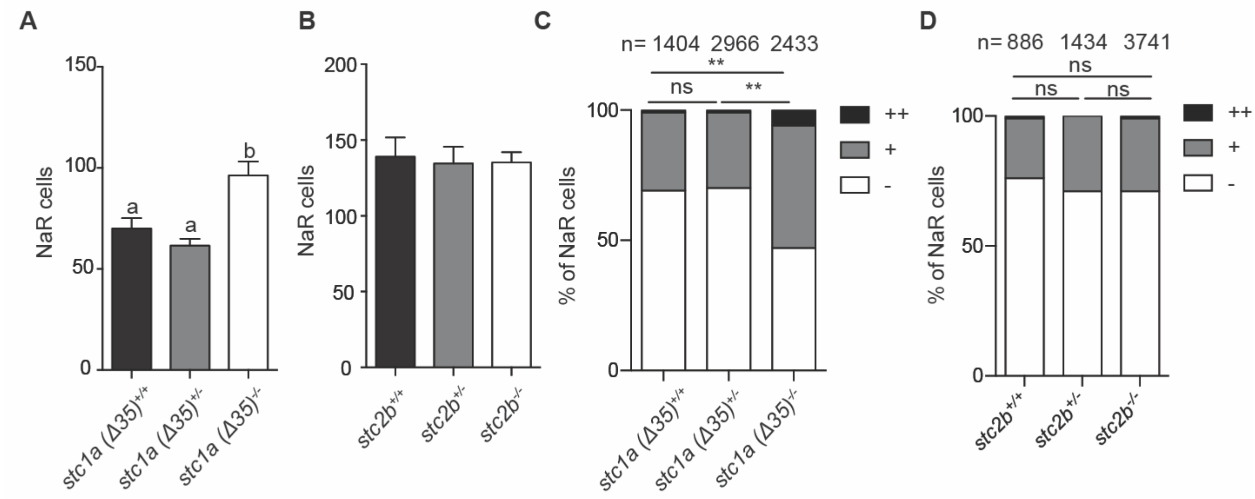


Supplemental Figure 4. Loss of Stc1a but not Stc2b increases NaR cell proliferation. (A-B) Progeny of *stc1a* (*Δ35*)$^{+/-}$ intercrosses (A), or *stc2b*$^{+/-}$ intercrosses (B) were raised and NaR cells were quantified as described Figure 3. n = 6 - 48 larvae/group. (C-D) NaR cell proliferation in 5 dpf larvae of the indicated genotypes were scored as described in Figure 3. **, $P < 0.01$ by Chi-square test. Total number of cells is shown above the bar.
